# Incidence and risk factors for cardiac arrest following upper cervical spine fractures in the elderly after minor trauma

**DOI:** 10.1007/s00402-026-06230-6

**Published:** 2026-02-19

**Authors:** Nils Mühlenfeld, Hans-Jörg Busch, Dominik Stephan, Benjamin Erdle, Andreas Frodl, Peter Obid, Hagen Schmal, Ferdinand C. Wagner

**Affiliations:** 1 Department of Orthopedics and Traumasurgery, University Medical Center Freiburg, Freiburg, Germany; 2 Emergency Department, University Medical Center Freiburg, Freiburg, Germany; 3 Department Of Orthopedic Surgery, Odense University Hospital Denmark, Odense, Denmark

**Keywords:** Cardiopulmonary arrest, Resuscitation, Cervical spine injury, Odontoid fracture

## Abstract

**Introduction:**

Cardiac etiologies are one of the main causes for out of hospital cardiopulmonary arrest (OHCA). Other etiologies might be easily overlooked, leading to delayed causal treatment. One such representative is neurogenic shock following upper cervical spine fractures after minor impact trauma in the elderly with little data on prevalence and risk factors, a drawback that we address in the present study.

**Material and Methods:**

Between 01/2013 and 12/2023, all patients ≥ 65 years with a fracture of the first and/or second vertebra after minor impact trauma were retrospectively reviewed to evaluate the incidence and character of OHCA. Resuscitated and non-resuscitated patients were compared regarding risk factors such as age, BMI, fracture classification and displacement, cardiac illness, laboratory parameters and bone mineral density.

**Results:**

Final data included 141 patients, 82.5 ± 7.6 years; 73 female and 68 male. The OHCA incidence was 9.9%. Pulseless electrical activity (PEA) (71,4%) and asystole (28.6%) were the only initial cardiac rhythms in these resuscitated patients. Patients were resuscitated for 14.4 ± 9.5 min before the return of spontaneous circulation (ROSC). The in-hospital survival rate was 21.4% (n = 3). Younger patient age (OR0.992 per year), a Anderson type II odontoid fracture (OR 1.145), as well as trauma during nighttime (6 pm–7am) significantly predicted OHCA (p < 0.05). Sex, BMI, bone density, fracture displacement and laboratory parameters (serum electrolytes (Sodium and Potassium), Lactate, pH, Hemoglobin, and Creatinine) revealed no direct impact on the likelihood to suffer from OHCA.

**Conclusions:**

With an occurrence of almost 10%, OHCA resulting from an upper cervical spine injury following low impact trauma is frequent in the elderly patient, and appears to be associated with non-shockable primary rhythms (PEA or asystole). If cardiac output is successfully restored, clinicians should always exclude an upper cervical spine fracture (especially an odontoid fracture). We therefore recommend an interdisciplinary primary assessment and cervical spine immobilization.

## Introduction

Over the last decades, interdisciplinary and centralized emergency departments have been implemented in Germany to improve timely, high quality medical treatment of patients with increasingly complex comorbidities due to an ageing society. Emergency medicine has therefore witnessed enormous progress, and departments are undergoing further professionalization [1]. This has led to the integration of interdisciplinary teams in acute emergency, where the patient is treated in a holistic approach while new and interdisciplinary methodologies are developed.

Out-of-hospital cardiac arrest (OHCA) remains a devastating medical condition leading to low survival rates and poor outcome. Treatment after cardiac arrest has become more complex over the last few years, which is why systematic efforts are being investigated to improve diagnostic- and treatment-algorithms [2]. Interestingly, the latest research suggests that “cardiac” etiologies may be the cause for cardiopulmonary arrest (CPA) less frequently than previously thought [3]. This might be of special interest with elderly patients, as they are more prone to critical illness and cardiac arrest than the remaining adult population [2, 4–7].

To emphasize the public health significance of this complex association, it is essential to examine its underlying epidemiological burden. The yearly occurrence of out-of-hospital cardiac arrest (OHCA) in Europe and North America ranges from 67 to 170 per 100,000 person-years, with survival rates to hospital discharge frequently under 10% [8, 9]. Moreover, Osteoporosis (OP) is the predominant bone disorder globally, affecting 200 million women worldwide and causing more than 8.9 million fractures annually [10], and is anticipated to rise substantially with the aging population. The significant prevalence of osteoporosis, especially among the elderly, contributes to the occurrence of cervical spine fractures, which manifest at a rate of 4–17 per 100,000 year [11, 12]. OP is characterized by a decrease in bone mass and deteriorated bone tissue and architecture, all of which raise the risk of fracture [13]. Because of the high incidence of OP combined with neurological disorders in the elderly, minor impact trauma often results in cervical spine injury in this age group [14–17]. Cervical spine injuries resulting from trauma with low impact kinetics have recently been reported to result in CPA in several low-number case reports [18–20]. The disruption of the supraspinal sympathetic pathways after trauma leads to parasympathetic dominance, and is considered the main cause of cardiovascular instability [21, 22]. Cardiovascular deficits including severe bradycardia, asystole and the loss of peripheral vascular tone are known complications [23]. Moreover, two recent studies reported that almost all patients with a cervical spine injury develop bradycardia, whereas dysfunction usually begins within 3–5 days after injury [24, 25]. Despite knowledge of this relation, fractures of the cervical spine are usually overlooked as the cause of OHCA [20]. Since acute cardiopulmonary deficiency in relation to an upper cervical spine injury has not been specifically evaluated in larger case studies, emergency physicians tend to neglect cervical spine injuries when the trauma likelihood in an elderly patient with cardiac arrest is unclear [18, 26]. Understanding the epidemiological background of this connection could be essential for the interdisciplinary emergency unit team to avoid a missed diagnosis and to develop treatment guidelines for the acute case of emergency.

The primary objective of this retrospective study therefore was to determine the epidemiology of OHCA in combination with fractures of the upper cervical spine in a large case series to evaluate whether cervical spine injuries should be considered in the diagnostic work-up of elderly patients over 65 years of age with OHCA after suspected minor impact trauma. Our secondary aim was to identify risk factors for the development of CPA in the elderly patient following cervical spine injury resulting from minor impact trauma.

## Patients and methods

This study followed the STROBE guidelines for observational studies (Strengthening the Reporting of Observational Studies in Epidemiology) and the RECORD guidelines (Reporting of studies Conducted using Observational Routinely collected Data) [27, 28].

A retrospective review was performed on a consecutive cohort of patients admitted to the emergency department of the University Hospital of Freiburg between January 2013 and December 2023. Inclusion criteria were patient age ≥ 65 years, a recent fracture of the first and/or second vertebra (C1 ± C2) diagnosed via computer tomography (CT) as well as a documented ground level fall or comparable low impact trauma. A “documented ground level fall” was defined as a fall from a standing height or less. “Comparable low impact trauma” included any mechanism with kinetic energy equivalent to or less than a ground level fall, such as a simple slip, trip, or a minor impact during activities of daily living. Patients were identified via a retrospective query of the hospital’s electronic medical records using the International Classification of Diseases Version 10 (ICD-10) codes of the German Diagnosis Related Groups (G-DRG). Patients’ characteristics, information and details about OHCA, disease-specific information, radiologic characteristics were abstracted and transferred to an electronic spreadsheet. Exclusion criteria were a high impact trauma mechanism, polytraumatized patients, concomitant injuries potentially the leading cause of OHCA as well as tumor disease, patients with a stroke or epilepsy, cardiac- or pulmonary embolism. Data sets containing inconclusive documentation as well as patients with falsely coded diagnoses were also excluded.

Two groups were formed after applying our inclusion and exclusion criteria:

1. “CPR”: Patients with a high cervical fracture (C1 ± C2) in whom OHCA associated with trauma was documented.
2. “nonCPR”: Patients with a high cervical fracture (C1 ± C2) in whom no OHCA associated with trauma was documented.

Time to return of spontaneous circulation (ROSC) after cardiopulmonary resuscitation (layman and professional time combined), amount of intravenous adrenaline, as well as the initial cardiac rhythm were extracted. Groups were compared regarding risk factors such as gender, BMI, age, fracture location and displacement, fracture classification, cardiac pre-existing conditions and their related pre-medication. Laboratory parameters (serum electrolytes, lactate, pH, hemoglobin, creatine) were compared and differences statistically evaluated as well. We specifically focused on Sodium and Potassium levels, in addition to markers of perfusion (Lactate, pH) and general status (Hemoglobin, Creatinine), as potential alterations in these electrolytes can occur during acute neurogenic shock and resuscitation, offering immediate clinical relevance. Atlas fractures (C1) were classified according to Gehweiler systematics [29]. Odontoid fractures (C2) were classified using the division introduced by Anderson and D’Alonzo [30] and C2 base or traumatic spondylolisthesis was evaluated using the Effendi classification [31]. All fractures were classified by two experienced trauma consultants. To evaluate bone density, CT morphological spongiosa density in form of Hounsfield Units (HU) was measured in line with previous research [32, 33]. Laboratory parameters (serum electrolytes, lactate, pH, hemoglobin, creatine) were compared and differences statistically evaluated as well.

### Statistical analysis

All variables were evaluated for normality distribution using a combination of histograms, quantile–quantile (Q–Q) plots and Shapiro–Wilk tests. Descriptive statistics were summarized as means and standard deviations for quantitative variables and as counts and frequencies for categorical variables across all variables. Incidences were evaluated using Chi-square test. Risk factors were compared using unpaired t-test or Mann–Whitney-U test depending on normality distribution. A linear regression was chosen to identify multiple independent variables. We additionally calculated the Odds Ratio (OR) for each independent predictor to quantify the magnitude of the association with OHCA. Statistical tests were calculated two-tailed using a 95% confidence level. Statistical significance was set at p < 0.05. Statistical analyses were performed with Graphpad Prism 10 (Graphpad, CA, San Diego).

### Compliance with Ethical Standards

Approval from our institutional review board was obtained before performing this retrospective study (**24–1014-S1-retro**). The study was performed in accordance with the ethical standards of the institutional and national research committee and with the 1964 Helsinki declaration and its later amendments. This research did not receive any specific grant from funding.

Informed consent was not obtained as a study was done in an anonymized retrospective manner.

## Results

### Sociodemographic data

**Fig. 1:**
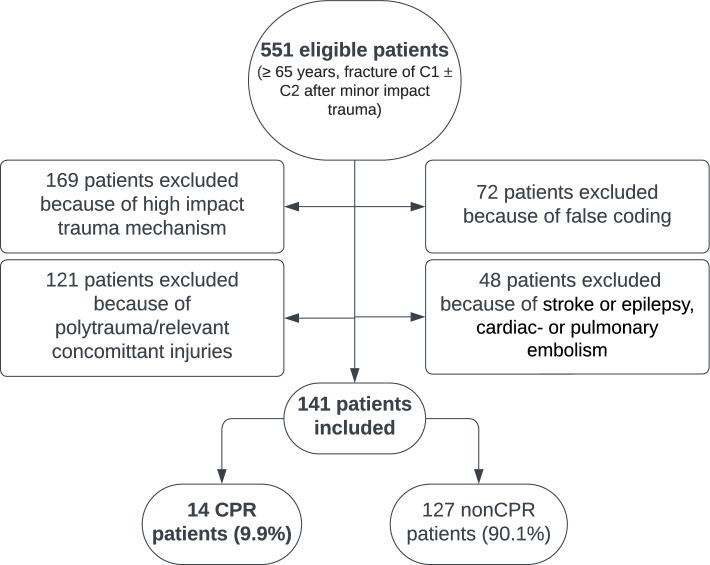
Patient flow chart to illustrate the eligibility process for data inclusion in this study. Out of 551 eligible patients age ≥ 65 years, after applying exclusion criteria, 141 patients could be identified for final data analysis and inclusion in this study. Of these, **9.9%** suffered OHCA after minor trauma due to the consecutive fracture of the first and/or second vertebrae

During January 2013 and December 2023, 141 patients met our inclusion criteria (Fig. 1). All data followed normal distribution. OHCA in relation to a high cervical fracture was documented in 14 patients (**9.9%**) (CPR patients). No OHCA prior to admission (nonCPR patients) was reported in 127 patients (90.1%).

Mean age of the final data set was 82 ± 5.2 years (95%CI: 81.3—83.8, Range: 65—101). Mean age for CPR patients was 78.7 ± 8.3 years (73.9 – 83.5, Range: 65 – 93) and 82.9 ± 7.4 years (81.6 – 84.3, Range: 66—101) for nonCPR patients respectively. Differences in age between the two groups were statistically significant (p < 0.048).

There were 51.8% (n = 73) female and 48.2% (n = 68) male patients in our cohort. In the CPR group 42.9% of patients (n = 6) were female and 57.1% (n = 8) were male, while in the nonCPR group 52.8% of patients (n = 67) were female and 47.2% (n = 60) were male. There was no significant difference in gender distribution between CPR and nonCPR patients (p = 0.482).

**Fig. 2:**
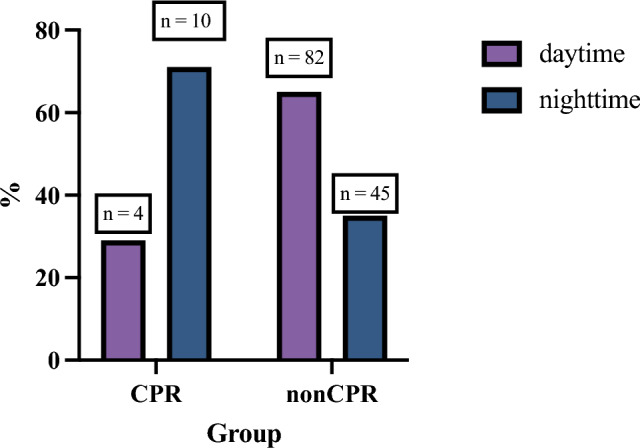
Time of Admission (daytime: 7am – 6 pm; nighttime: 6 pm – 7am) for CPR and nonCPR patients in %. Differences between groups were statistically significant (p = 0.018)

Regarding the time of admission, 71.4% (10/14) of CPR patients presented in the emergency department during nighttime (6 pm – 7am), while in the nonCPR group only 35.4% (45/127) of patients presented during nighttime (Fig. 2.). This difference was statistically significant (p = 0.018).

### Character of OHCA, return of spontaneous circulation (ROSC) and survival rate

Out of the 14 CPR patients that presented after OHCA, 71.4% (n = 10) did so with an initially documented cardiac rhythm of pulseless electric activity (PEA), while 28.6% (n = 4) initially presented with asystole. Shockable initial rhythms like ventricular fibrillation and chamber flutter were not observed in any of the patients with OHCA following an upper cervical fracture. Patients were resuscitated for a mean of 14.4 ± 9.5 min (95% CI: 8.7 – 20.1; range: 3 – 30) prior to ROSC (sum of layman and professional resuscitation). The entire cohort’s in-hospital survival rate was 90.1% (n = 172). Survival rate in the CPR group was 21.4% (n = 3) and 97.6% (n = 124) in the nonCPR group.

### Risk factors, fracture distribution and classification

Independent risk factors to suffer OHCA in relation to a fracture of the upper cervical spine after minor trauma in the elderly were evaluated. Sex, BMI, fracture dislocation, bone density, and the evaluated lab parameters revealed no risk factor (Table 1**.**).

**Table 1:** Potential risk factors analyzed in their different values between patient groups. Values are reported in mean ± SD as are their values of significance

Risk factor	CPR	nonCPR	p
Age (years)	78.7 ± 8.3	82.9 ± 7.4	0.048*
BMI	26.5 ± 4.9	26.8 ± 5.3	0.739
Fracture dislocation (mm)	4.3 ± 3.9	3.2 ± 3.0	0.439
Hounsfield Units (U)	254.9 ± 85.4	229.5 ± 71.7	0.236
Creatinine (mg/dl)	1.2 ± 0.4	1.2 ± 0.9	0.207
Hemoglobin (g/dl)	12.0 ± 2.4	12.7 ± 1.8	0.213
Sodium (mmol/l)	140.3 ± 3.8	138.4 ± 4.5	0.130
Potassium (mmol/l)	4.3 ± 0.6	4.4 ± 0.7	0.735

*Statistically significant

Documentation regarding preexisting cardiac conditions prior to admission were inconclusive or insufficient in the CPR-patient group due to their high rate of exitus letalis. We could therefore not assess this specific risk factor further. Regarding the distribution of fracture type overall, however, we noted a statistically significant difference (p = 0.003). Here we observed that an Anderson II odontoid fracture was the predominant type with 92.8% (13/14) in the CPR group and only 59.8% (76/127) in the nonCPR group (p = 0.018). Furthermore, all patients (14/14) in the CPR group presented with an odontoid fracture of any type, while this was observed in only 71.6% (101/127) of nonCPR patients (p = 0.019). Traumatic spondylolysis of C2 (Effendi fractures) were recorded in 7.1% (9/127) of nonCPR patients and in 0.0% (0/14) of CPR patients, respectively (p = 0.599) (Table 2).

**Table 2:** Distribution of fracture type, portrayed with their respective numbers and percentages (some patients had suffered multiple fractures)

Fractured Vertebra	Fracture Classification	CPR n (%)	nonCPR n (%)
Atlas (C1)	Gehweiler I	1 (7.1)	7 (5.5)
	Gehweiler II	1 (7.1)	14 (11.0)
	Gehweiler III	2 (14.3)	7 (5.5)
	Gehweiler IV	0 (0.0)	2 (1.6)
	Gehweiler V	0 (0.0)	0 (0.0)
Odontoid (C2)	Anderson/D’Alonzo I	0 (0.0)	2 (1.6)
	Anderson/D’Alonzo II	13 (92.8)	76 (59.8)
	Anderson/D’Alonzo III	1 (7.1)	23 (18.1)
Traumatic	Effendi I	0 (0.0)	4 (3.2)
spondylolysis	Effendi II	0 (0.0)	5 (3.9)
(C2)	Effendi III	0 (0.0)	0 (0.0)

We conducted multiple linear regression to test whether patient age, a type II Anderson odontoid fracture, fracture dislocation, and/or patient sex significantly predicted OHCA. The overall regression was statistically significant (R2 = 0.78, F(2.865) = 12.60, p = 0.026). We found that younger patient age and a type II Anderson odontoid fracture significantly predicted OHCA. Fracture dislocation and sex did not significantly predict OHCA (Table 3.)

**Table 3:** Multiple regression revealed younger patient age and a type II Anderson odontoid fracture as independent predictors for OHCA in our cohort

Model	Regression- coefficient	SD	Odds Ratio (OR)	95% Confidence Interval (CI)	Significance (p)
Constant	1.500	0.308			< 0.001
Patient age	-0.008	0.003	0.992	0.986–0.998	0.028*
Anderson II	0.135	0.054	1.145	1.030–1.272	0.013*
Fracture dislocation	-0.003	0.008	0.997	0.981–1.013	0.705
Sex	0.007	0.052	1.007	0.909–1.115	0.899

* Statistically significant

Note on Patient Age: Patient age was included in the multiple regression as a continuous variable, with the negative coefficient (B =  − 0.008) indicating that increasing age is associated with decreased odds of OHCA. No specific age cutoff was used in this multivariate model; however, descriptive analysis showed the mean age of CPR patients (78.7 ± 8.3 years) was significantly lower than nonCPR patients (82.9 ± 7.4 years)

## Discussion

The most important finding of this study is that the OHCA occurrence in elderly patients aged over 65 years with an upper cervical spine injury after minor impact trauma is higher than expected (almost 10% in this analyzed cohort). Considering the numerous seniors with prehospital death after low impact trauma consequently leading to severely underreported OHCAs following high cervical fracture, we can carefully consider this percentage to be substantially higher [2, 34–36]. OHCA resulting from an upper cervical spine injury after a fall is therefore not a rare occurrence at all in the elderly, and it should always be taken into account, when the OHCA cause is unclear. After suspected low impact trauma and consecutive OHCA, admitting such patients to a hospital equipped with an interdisciplinary team of emergency physicians and specialized orthopedic/trauma surgeons is therefore strongly recommended. This position is in line with the German resuscitation council, which recommends optimizing in-hospital acute care after a successful resuscitation in form of an interdisciplinary trained admission team designated as a cardiac arrest receiving team (CART). The CART’s objective is to provide primary care to resuscitated patients in a standardized manner and relying on predefined diagnostic and therapeutic pathways including trauma pathways [37, 38].

Interestingly, we found in this study that younger patient age and a type II Anderson odontoid fracture predicted OHCA significantly, while fracture dislocation and sex did not. All resuscitated patients (14/14) presented with an odontoid fracture upon admission, a factor that differed significantly from the non-resuscitated patients. Above all, at 92.8% (13/14), the rate of Anderson Type 2 odontoid fractures is particularly high among CPR patients. Traumatic spondylolysis of C2 (Effendy fractures) were rarely observed after a low impact trauma in the elderly (9/141 patients, 6.4%) and did not result in OHCA in the present cohort (0/14 CPR patients) which might be explained by the high energy needed to result in such “hangman’s fractures”[31]. Nevertheless, immobilization of the cervical spine should be considered unless it results in postponing appropriate intubation and resuscitation [39]. Data from this study support the demand for improved teamwork and the expertise of interdisciplinary teams in the emergency department [40–42]. Interestingly, most of the resuscitated patients were admitted during nighttime (6 pm – 7am), which did not hold true for our nonCPR patients. In case of CPA after a minor fall, teams of doctors of internal medicine and trauma-surgeons should therefore be available on short notice and consider the strong possibility of the presence of an upper cervical injury [43].

While one cannot definitively ascertain in each case whether the cardiopulmonary arrest was the initial cause for the fall triggering a cervical spine injury or if it was the reverse, Turnham et al. proposed that cervical spine fractures after a cardiac arrest are extremely rare, and that it is the injury itself that usually triggers the cardiac arrest [19]. Additionally, Tam et al. evaluated in 2023 via full body CT-scans the concomitant injuries in patients who had been resuscitated and found cervical spine injuries to be rare occurrences, that is, in less than 2% of such patients [43].

**Fig. 3:**
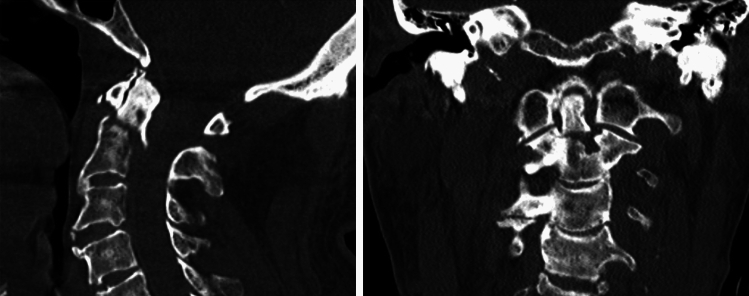
Sagittal and coronary CT view of an 89-year-old male who suffered OHCA in relation to a fresh Odontoid “Anderson II” fracture after a ground level fall. Initial cardiac rhythm was PEA. ROSC was reached after a total of 25 min of CPR (layman and professional CPR combined) and the admission of 1 mg of intravenous adrenaline. Exitus letalis occurred within 24 h in the intensive care unit

A trauma-induced cardiovascular deficit evolves by the disruption of supraspinal sympathetic pathways. Through the consecutive dominance of the parasympathetic system, bradycardia and a loss of peripheral vascular tone occur immediately or a few days after a cervical spine injury, which can lead to cardiovascular instability and asystole [21, 22, 24, 25]. Our data support this pathophysiology, as PEA and asystole were the only initially documented cardiac rhythms in resuscitated patients (71,4% vs. 28.6%), while ventricular fibrillation and chamber flutter were not observed in any patient. The presence of PEA or asystole in a patient after a minor fall might therefore raise the probability for a cervical spine injury, whereas in elderly patients presenting ventricular fibrillation or chamber flutter, a cervical spine injury seems unlikely to be the cause of CPA. Moreover, most patients returned to spontaneous circulation after the administration of one or two milligram of intravenous adrenaline as part of the resuscitation algorithm. This can be attributed to pharmaceutical support of failing sympathetic pathways following neurologic shock. Early administration of intravenous adrenalin should therefore be of high priority in the resuscitation of elderly patients when minor trauma is suspected (Fig. 3).

The measurement of Hounsfield Units (HU) seems to be a suitable tool to readily and accurately assess bone quality in vertebrae bodies with high inter-rater reliability [44–46]. We identified no relevant difference in HU between the CPR and nonCPR patients in this study, which indicates that higher bone density does not seem to be a protective factor against OHCA after cervical spine injury in the elderly. Nevertheless, OP raises the likelihood of sustaining fractures after a minor fall in the elderly [14, 15, 47], and the present data demonstrate high probability to suffer OHCA as a direct result of a cervical spine fracture after minor trauma (9.9%), it indirectly raises the risk for OHCA in comparison to the remaining population without OP. OP-therapy is therefore an essential remedy to help prevent OHCA after a minor trauma fall in the elderly. Fall-related fractures should be considered a red flag when reviewing a patient’s individual therapeutic regime regarding OP as well as potentially underlying neurological disorders like Parkinson’s Disease that can promote OP and increase the risk of falling [16, 17, 47]. Elderly patients with a falling tendency or an increasing fall-rate should always be checked for prevalence of osteoporosis.

From the data we present here, a patient age below 80 years seems to be a risk factor to suffer OHCA after an odontoid fracture resulting from minor trauma. One likely explanation is however, that out of hospital CPA in patients aged over 80 years tends to be terminated early more often or not even initiated by prehospital emergency teams because survival rates after OHCA are proven to be reduced with increasing age [34, 48]. This factor could have led to a lower hospital admission rate in this cohort.

Furthermore, we were unable to identify any other independent risk-factor for OHCA in connection with an upper cervical spine injury after minor trauma. Gender, fracture dislocation, BMI and blood values (creatinine, hemoglobin, electrolytes) demonstrated no verifiable impact on the likelihood to suffer OHCA in the 65-year-old and older patient after minor impact trauma. Unfortunately, our documentation of preexisting cardiac conditions prior to admission was inconclusive or -insufficient in the CPR-patient group because of their high rate of exitus letalis, which meant that this specific risk factor could not be evaluated.

### Limitations

Some limitations must be considered for this study, starting with its retrospective design. Second, patients in the two evaluated groups might have had different characteristics that were not screened. A treatment algorithm cannot be drawn from the results of this study.

No clinical outcome other than the survival rate was evaluated. We are however the first to describe a larger number of consecutive cases of elderly patients who suffered OHCA following an upper cervical spine injury in connection with low impact trauma. Further larger prospective studies reporting on mid-term and long-term as well as clinical outcomes are necessary to further evaluate this pathophysiological pathway and improve the treatment of such elderly patients.

## Conclusions

With an occurrence of almost 10%, OHCA resulting from an upper cervical spine injury following low impact trauma is frequent in the elderly patient, and appears to be associated with non-shockable primary rhythms (PEA or asystole). If cardiac output is successfully restored, clinicians should always exclude an upper cervical spine injury (especially an odontoid fracture). We therefore recommend an interdisciplinary primary assessment and cervical spine immobilization.

## Data Availability

No datasets were generated or analysed during the current study.
